# Inline Quality Control through Optical Deep Learning-Based Porosity Determination for Powder Bed Fusion of Polymers

**DOI:** 10.3390/polym14050885

**Published:** 2022-02-23

**Authors:** Samuel Schlicht, Andreas Jaksch, Dietmar Drummer

**Affiliations:** 1 Institute of Polymer Technology, Friedrich-Alexander-Universität Erlangen-Nürnberg, Am Weichselgarten 10, 91058 Erlangen, Germany; lkt-info@fau.de; 2 Collaborative Research Center 814, Friedrich-Alexander-Universität Erlangen-Nürnberg, Am Weichselgarten 10, 91058 Erlangen, Germany; andreas.jaksch@fau.de

**Keywords:** powder bed fusion, selective laser sintering, deep learning, deep neural network, process monitoring, quality control

## Abstract

Powder bed fusion of thermoplastic polymers is a powder based additive manufacturing process that allows for manufacturing individualized components with high geometric freedom. Despite achieving higher mechanical properties compared to other additive manufacturing processes, statistical variations in part properties and the occurrence of defects cannot be avoided systematically. In this paper, a novel method for the inline assessment of part porosity is proposed in order to detect and to compensate for inherent limitations in the reproducibility of manufactured parts. The proposed approach is based on monitoring the parameter-specific decay of the optical melt pool radiance during the melting process, influenced by a time dependency of optical scattering within the melt pool. The underlying methodology compromises the regression of the time-dependent optical melt pool properties, assessed in visible light using conventional camera technology, and the resulting part properties by means of artificial neural networks. By applying deep residual neural networks for correlating time-resolved optical process properties and the corresponding part porosity, an inline assessment of the spatially resolved part porosity can be achieved. The authors demonstrate the suitability of the proposed approach for the inline porosity assessment of varying part geometries, processing parameters, and material aging states, using Polyamide 12. Consequently, the approach represents a methodological foundation for novel monitoring solutions, the enhanced understanding of parameter–material interactions and the inline-development of novel material systems in powder bed fusion of polymers.

## 1. Introduction

Laser-based powder bed fusion of polymers (PBF-LB/P) is a powder-based additive manufacturing process of polymer components that is subject to a variety of process influences. These include exposure parameters [1], varying geometries [2], and physical as well as chemical properties of the applied material [3]. Given the significant process variance observed in the powder bed fusion of polymers and the influence of varying boundary conditions, a process-integrated framework for assessing part-specific properties is required. Mechanical properties of parts produced through PBF significantly depend on the occurrence of pores. Therefore, the formation of porous regions represents a critical issue in PBF-LB/P. To overcome the issue of a limited understanding of the relations between part properties and the underlying process, the monitoring and subsequent modeling of these aspects constitutes a critical factor for enhancing the reproducibility of PBF-LB/P. Given the complexity of the process, traditional regression models are limited in handling the inherent variance and the complexity of underlying interdependencies. In contrast to traditional approaches, artificial neural networks have proven to model highly complex relations that lack an explicit analytic description. Therefore, deep artificial neural networks represent a possibility to model the complex relations between process properties and the resulting part properties in PBF-LB/P.

### 1.1. Fundamental Process Influences in the Powder Bed Fusion of Polymers

Laser-based powder bed fusion of polymers is characterized by the iterative execution of the three consecutive process steps powder coating, laser-based exposure, and the subsequent consolidation of the molten polymer [4]. Resulting part properties are influenced by underlying boundary conditions, such as the part geometry [2,5], the geometry-dependent layer time [6], and various process parameters, such as applied exposure parameters [1,7,8]. With regard to the exposure step, extensive research has been applied towards the influence of exposure parameters and exposure strategies to define optimal parameters. Given the influence of the exposure process, an interaction between exposure parameters and the exposed geometry was observed by Greiner et al. (2020) [9] and Wegner et al. (2015) [8]. The authors describe a negative correlation of the spatial expansion of consecutive scan paths and the resulting melt-temperature, thus implying an interdependence of the part geometry and the applied scanning parameters on part properties. Further time-dependent influences [6,10] of the scanning step are given by varying layer times, described by Pavan et al. (2017) [6], indicating a positive correlation of increased layer times and the corresponding mechanical properties. Drexler et al. (2015) identified an influence of the exposure speed on mechanical part properties [10], which can be correlated with a reduced thermal penetration depth at increased exposure speeds. Further interdependencies between material properties, processing parameters, and resulting part properties can be derived with regard to the used material. Kruth et al. (2007) [11] demonstrated the influence of rheological material properties on the consolidation step. Considering the use of pre-used polymers powders, as described by Wudy et al. (2019) [12] and Chen et al. (2018) [13], the influence of aging-induced varying rheological material properties can lead to significant shortcomings in part properties, affecting both mechanical part properties [3] and microstructural properties [14]. Due to altered crystallization properties [15] and increased particle sizes [16] of aged powders, material aging is inherently connected to varying processing conditions. The influences mentioned above and interdependencies between processing parameters and boundary conditions represent a major issue towards the analytical optimization and the reproducibility of part properties. These shortcomings indicate the necessity for a comprehensive monitoring approach to account for statistical variations in the processing behavior and the resulting part properties.

### 1.2. Monitoring Approaches in the Powder Bed Fusion of Polymers

In order to enhance the understanding of underlying physical mechanisms and to enable the monitoring of the powder bed fusion process, several approaches have been proposed. Thermographic approaches for monitoring PBF have been demonstrated for use in metal-based PBF [17,18,19] and polymer-based PBF [8,20,21]. Thermographic monitoring allows for the in situ measurement of resulting temperature fields, thus providing meaningful insights into the melting process. Therefore, thermographic approaches allow for the in situ identification of defects and the enhanced understanding of the influence of processing parameters on thermal process conditions [8,9,20] and fundamental investigations of PBF-LB/P [22]. In addition to thermographic monitoring, the application of optical coherence tomography for the process-integrated measurement of geometric melt pool properties [23,24] and the inline detection of defects [25] has been proposed. In contrast to thermographic process monitoring, optical coherence tomography allows for the three-dimensional analysis of the powder bed and the melting process.

Summarizing existing monitoring approaches, monitoring of PBF-LB/P predominantly focuses on the thermographic and optical investigation of the energy input. Although the consolidation step, characterized by the viscous flow of the material [26,27], accounts for most of the processing time, existing approaches for the monitoring of PBF-LB/P do not comprehensively address the consolidation phase and resulting part properties.

### 1.3. Machine Learning in the Context of Additive Manufacturing

The application of machine learning for modeling complex dependencies in powder bed fusion, specifically of polymers, has been adopted in various applications [28]. Applications of machine learning include the post-processing of computed tomographic investigations [29], the prediction of mechanical properties based on parameter sets [30] and the classification of process states [31]. Given the complex dependencies observed in PBF-LB, both metal- and polymer-based, defects cannot be avoided systematically [31]. Recently, emerging research on the monitoring of laser-based additive manufacturing processes focuses on the classification of process states [31] and the detection of defects within the process [32,33,34,35,36]. Existing approaches for the inline quality control of laser-based powder bed fusion apply image processing for detecting defects within distinct layers [32,33,35,36] based on optical [31,36] and thermographic [32] imaging. The underlying methodology predominantly relies on the application of convolutional neural networks (CNN) to process static image information. In contrast to the predominant focus on static analysis in recent research, Yuan et al. (2019) [37] describe the CNN-based monitoring of metal-based PBF by analyzing time-resolved video data. Other approaches, that differ from the application of convolutional neural networks, include the use of k-means clustering, described by Grasso et al. (2017) [38] and the application of Bayesian classifiers for the classification of visual images, proposed by Aminzadeh et al. (2019) [31].

Zhang et al. (2019) [39] proposed using on-axis imaging for the inline extraction of porosity data, extending the mere classification of defects towards a quantitative approach by predicting spatially resolved porosity values for PBF-LB of metal alloys. In contrast to imaging-based methods, Shevchik et al. (2018) [40] demonstrated the deep learning-based analysis of time series based on acoustic emissions in PBF-LB of stainless steel. A similar approach has been employed by Ye et al. (2018) [41], thus enabling the identification of process defects using an acoustic analysis and the subsequent application of a deep belief network. Therefore, the deep learning-based analysis of time series embeds the potential of deriving quality metrics based on the temporal variability of signals measured within the process.

### 1.4. Neural Network-Based Modeling of Complex Dependencies

The use of artificial neural networks as a specific machine learning method has been proven especially useful for modeling complex dependencies when given sparse, high-dimensional datasets [42,43]. Deep artificial neural networks represent an effective way for handling the resulting so-called curse of dimensionality when modeling complex, multi-dimensional data [43]. In contrast to shallow networks, implementing deep artificial neural networks requires structural adoptions of the network architecture to avoid gradient vanishing [44]. To minimize the effects of gradient vanishing, He et al. (2016) proposed the integration of residual connections between non-consecutive layers within the structure of neural networks, thus enabling the efficient implementation of deep neural networks. Residual neural networks and related networks architectures have been widely adopted in image processing and represent the rapidly developing state of the art in image recognition [45,46,47]. Apart from a wide range of applications in image recognition and image super-resolution [48], the successful applicability of deep neural networks has been demonstrated for the classification [49] and the forecasting [50] of time series, constituting the technological basis of the present paper.

## 2. Materials and Methods

### 2.1. Conceptualization of Inline Porosity Monitoring

**Figure 1:**
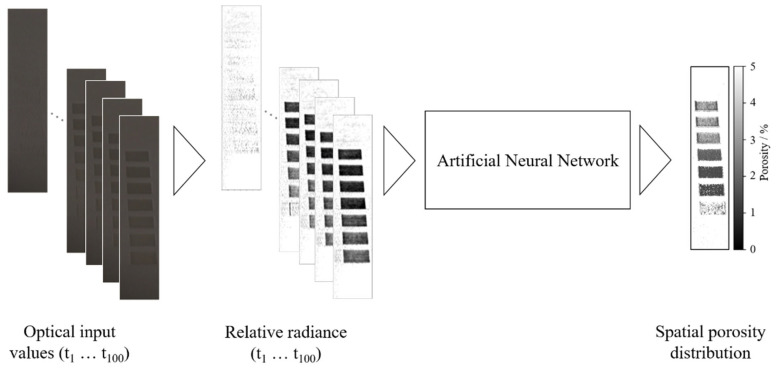
Illustration of the applied approach for deep learning-based process-property regression.

To date, the inline monitoring of the powder bed fusion of polymers is limited to the image-based detection and classification of defects. The presented approach aims to overcome the limitations of existing concepts by correlating optical data, derived by means of inline monitoring, and resulting part properties. The technological basis compromises the combination of time-resolved optical inline process monitoring and subsequent data processing using deep residual neural networks. The applied neural network is designed to model a regression of optical melt pool properties and the resulting, spatially resolved part porosity. The porosity of manufactured parts significantly influences the resulting part quality, as it directly affects resulting mechanical properties [51] and is, therefore, chosen as the metric for the inline assessment of part properties. The applied input dataset compromises a time series of consecutive scalar values, representing the time-resolved optical radiance of the powder bed within a particular area, e.g., a pixel. Following the processing within the network, a scalar output is received that represents the porosity of the particular input area within the monitored layer. The approach compromises the reduction from a three-dimensional, time-resolved dataset of optical values to a two-dimensional dataset representing spatially resolved porosity values of the monitored layer, shown in Figure 1.

**Figure 2:**
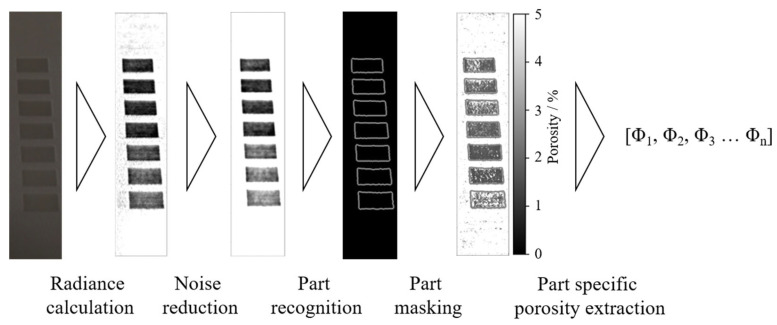
Schematic illustration of the process for obtaining layer- and part-specific porosity values.

Image post-processing of raw data, displayed in Figure 1, compromises calculating the radiance of each pixel relative to an unexposed area within the powder bed to compensate for temporal variations in the illumination of the powder bed surface. Based on the two-dimensional porosity matrix, obtained from the neural network, the proposed approach allows for the process-integrated, spatially resolved identification of defects. A subsequent identification of part-specific cross-sections allows for the inline determination of part properties, schematically illustrated in Figure 2.

For determining part-specific values Φ_n_, the contours of distinct cross-sections are detected using the Canny edge detector algorithm [52]. By combining layer-specific part-recognition and the previously obtained two-dimensional porosity matrix, porosity values corresponding to specific cross-sections can be derived automatically. By determining the weighted mean of layer-specific porosity values, part-specific values are obtained. Resulting layer-specific and part-specific porosity values represent the basis for a subsequent quality analysis based on pre-defined thresholds, corresponding to an acceptable porosity level. The artificial neural network, applied for the described inline monitoring, is trained by correlating part-specific optical data, obtained from experimental data acquisition, and experimentally obtained porosity values of the corresponding samples. With regard to the application of supervised learning, both optical data and corresponding porosity values need to be generated prior to the training process. Consequently, the proposed approach can be distinguished from existing approaches with regard to the integration of time-resolved data. In contrast to the application of convolutional neural networks, the porosity of a particular pixel is determined independent of surrounding pixels, relying on the temporal change of optical characteristics of each particular area.

### 2.2. Experimental Set-Up

All training data acquisition for the inline monitoring process is based on processing conventional, commercially available Polyamide 12 (PA 2200), supplied by EOS GmbH, Krailling, Germany. The training dataset is obtained using virgin powder with a viscosity number (ISO 307) of VN = 55 mL g^−1^. The physical properties of the applied virgin material are displayed in Table 1. Thermal characteristics are determined by means of differential scanning calorimetry, using a heating rate of 10 K min^−1^.

In addition to using virgin powder, the validation of the trained neural network is conducted using pre-aged PA 12. To ensure constant physical and chemical properties, virgin powder is aged at controlled conditions inside the build chamber by applying a powder bed surface temperature of 168 °C and a chamber temperature of 150 °C. The aging-induced change in physical properties is displayed in Table 2.

All experimental data acquisition is conducted using a PBF-LB/P system of the type EOS Formiga P110, equipped with a 30 W CO_2_-Laser. Given the pre-defined optical system, a focal diameter of 500 µm with a Gaussian intensity distribution is applied. For obtaining time-resolved optical data, the powder bed is monitored in visible light using a Sony IMX477 CMOS sensor, SONY Corporation, Tokyo, Japan and a f = 50 mm lens, equipped with an adjustable aperture. Recorded images are processed using bespoke hardware combined with a single chip tensor processing unit, supplied by Google LLC, Mountain View, California, to allow for the efficient inference of deep residual neural networks. 

The camera assembly is positioned and focused to cover a cross-sectional area of 115 × 150 mm^2^ on the powder bed surface. Considering the anisotropic properties of the radiance of the melt pool, a camera angle of 35° relative to the powder bed surface and further monitoring parameters, displayed in Table 3, are kept constant. With regard to the inherent perspective distortion induced by the camera angle, the spatial resolution represents an average value.

**Figure 3:**
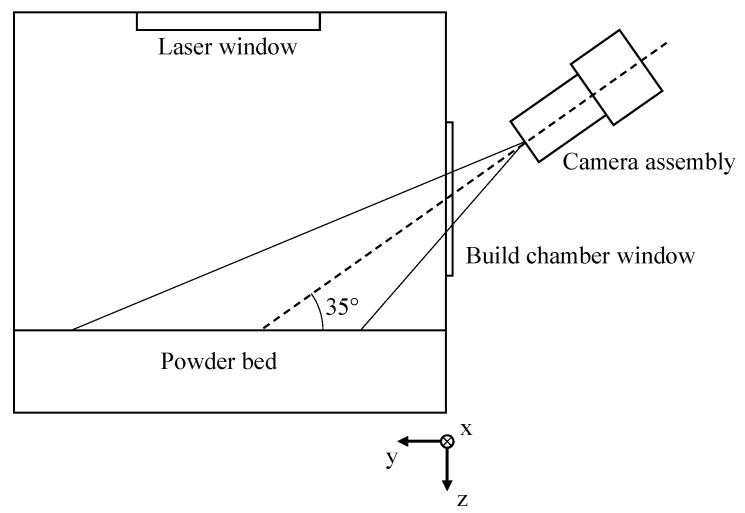
Schematic illustration of the applied setup for the optical monitoring of the powder bed surface.

To obtain a homogenous illumination, two black body radiators (T = 4000 K) installed inside the build chamber are used. The applied setup, consisting of a camera assembly and the monitored powder bed, is displayed schematically in Figure 3.

Subsequent numerical post-processing is based on RGB images, recorded using the bmp file format. The dataset of each recorded time step, obtained from the camera, is converted to a grayscale image in real-time. The calculation of optical powder bed properties relies on the spatially resolved radiance of the powder bed surface, L, relative to a reference value of the non-exposed powder bed, L_0_. The observed electromagnetic spectrum is limited to a range from 380 nm to 680 nm using an optical filter.

L_relative_ = L/L_0_ (1)

The normalized, dimensionless value is monitored over time using a frequency of 1 Hz, resulting in a time and spatially resolved dataset representing the input values of the neural network. Therefore, the time-dependency of the normalized radiance, referred to as relative radiance, constitutes the main metric for quantifying optical process properties.

### 2.3. Experimental Acquisition of Training Data

**Figure 4:**
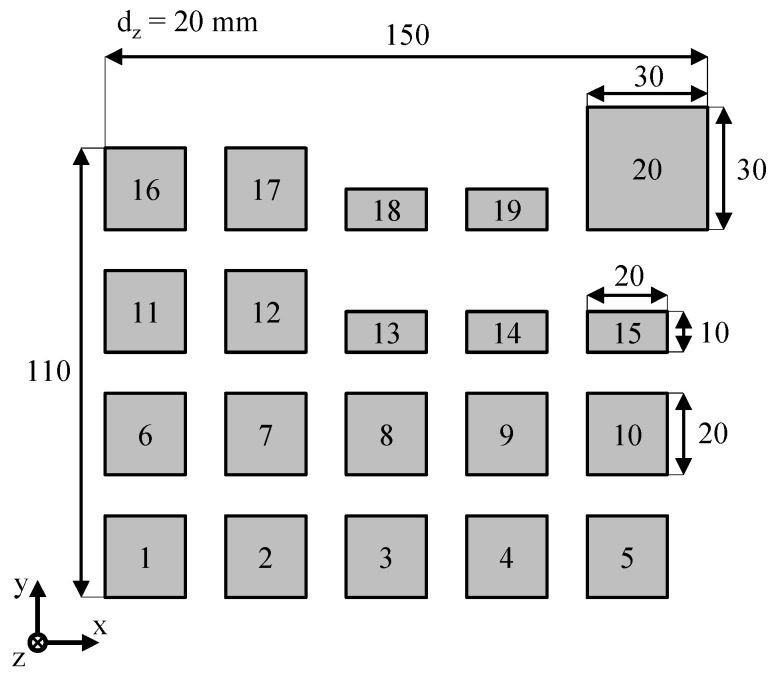
Build process layout for the generation of component-specific training data.

The dataset for training the neural network is obtained by varying the applied laser power, the exposure speed, and the hatch distance. Resulting optical, part-specific melt pool properties are subsequently correlated with experimentally derived values of the volumetric porosity. An increased variance of process properties is obtained by varying the exposed cross-sections, as shown in Figure 4.

To implicitly address the occurrence of interdependencies between varying process parameters, laser power, scan speed, and hatch distance are varied across a wide range. Underlying parameter combinations used for the generation of training data are displayed in Table 4. Applied boundary conditions include a powder bed surface-temperature of 168 °C, a build chamber temperature of 150 °C and a layer thickness of 0.1 mm. Alternating meander scanning is applied as the underlying exposure strategy. To obtain a sufficient number of training samples, the process layout is built and monitored twice to increase the number of samples and to implicitly integrate statistical variations of the building process into the dataset. Finally, the dataset used for training the neural network is obtained by correlating optical process properties of the last five layers of a particular part with the corresponding part-specific porosity. With regard to the cuboid shape of the training samples, part-specific volumetric porosity values Φ are assessed by combining gravimetric and volumetric measurements of the manufactured specimens by determining the sample mass m and the sample volume V with regard to the raw material density ρ_0_.

(2) $$\Phi =1\;-\frac{m}{V\;\rho_{0}}$$

Considering the monitoring of five consecutive layers of each build job, the applied dataset includes 200 samples, each compromising a component-specific optical time series and the corresponding, experimentally determined porosity value of the manufactured cuboid sample. Therefore, the optical representation of the consolidation, observed during the production of the final layers of the rectangular part, is correlated with experimentally obtained values for part-specific volumetric porosity values. In addition to the experimental measurement of part-specific samples, 20 training samples corresponding to non-exposed cross-sections of the powder bed are included in the dataset. All porosity values corresponding to non-exposed samples are set to a value of 56%, consistent to the relative bulk density of the powder. The resulting dataset corresponds to a two-dimensional array of part-specific time-resolved optical characteristics and corresponding part-specific porosity values.

Consequently, the applied process layout for the generation of training data covers a broad range of exposure parameters, predominantly focusing on the fabrication of cubic specimens.

### 2.4. Network Architecture and Implementation

**Figure 5:**
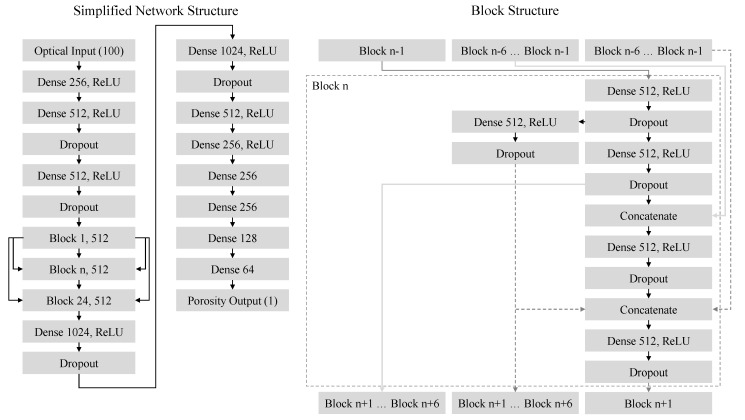
Schematic overview of the structure of the applied neural network with simplified block structures; Overview of the corresponding block structure, displaying connections to preceding and following blocks.

The residual artificial neural network, applied for the regression, is characterized by a segmented structure. This structure compromises an entry flow, a repetitive block structure and an exit flow. The entry flow receives 100 scalar values as input values, representing the normalized change of the radiance of a particular cross-section within the powder bed surface over time. After being processed by the residual block structure, a converging exit flow leads to the output of a single porosity value, corresponding to the porosity of the particular cross-section within the monitored layer. To increase the generalization capabilities of the neural network compared to a shallow network, a number of 24 consecutive blocks is applied. The resulting neural network compromises 131 fully connected layers, subsequently referred to as “Dense layers”, displayed in Figure 5. In addition to fully connected layers, the implementation of dropout layers [53] within the network further contributes to the generalization of the underlying dataset. To enhance the inherent feature extraction, the block structure compromises a complex information flow characterized by the implementation of three distinct information flows, shown in Figure 5. Each block structure receives information from up to six preceding blocks, thus significantly increasing the number of residual connections within the network. Given the fixed input of the applied network, compromising 100 consecutive time steps, the layer time is limited to a period of 100 s. Based on a predominant occurrence of layer times within a range of 20 s to 40 s, datasets with a duration below 100 s are synthetically enlarged by adding virtual time steps before the beginning of the layer. These virtual time steps correspond to the value of the first measured time step of the particular cross-section within a specific layer, indicating no change in optical properties before the beginning of the monitoring. Therefore, a vector of 100 float values, corresponding to the temporal change of the relative radiance of a particular cross-section, is applied as the input format.

Supervised training of the neural network is performed using backpropagation, specifically by applying the adaptive moment estimation [54] as the underlying optimizer. Boundary conditions and hyperparameters applied for training the neural network are summarized in Table 5.

The artificial neural network is implemented using the TensorFlow framework, Version 2.4.1. Keras 2.4.0 is applied as the main library for implementing the structure of the neural network.

Subsequent optimization of the applied artificial neural network is based on varying the number of neurons of each layer within the block structure. In order to identify an optimum width of the network, the number of neurons is varied across a broad range from 2 to 512, corresponding to a power function of natural numbers to the base of 2. Other elements and structures of the applied neural network remain unchanged during the optimization step. The structural optimization is conducted by training the neural network for consecutive 10,000 epochs. A randomized 80:20 split for training and validation of the network, respectively, is applied to assess the generalization capabilities of the neural network in dependence on the underlying number of neurons.

### 2.5. Validation of the Generalization Capability of the Trained Network

Validating the generalization capability of the trained neural network is essential for assessing the capability of the network to model part properties of a broad range of processing parameters, exposed cross-sections, and physical powder properties. The validation of the trained neural network is based on varying exposure parameters, the application of pre-aged Polyamide 12 powder and varying geometric boundary conditions. Varied exposure parameters are chosen according to a polynomial profile to evaluate the influence of slight and significant changes of process parameters on the prediction accuracy. Geometric deviations compared to the previous training process layout include the application of square cross-sections with an edge length of 10 × 10 mm^2^ and the application of thin-walled specimens to assess the influence of varying cross-sections on the generalization capabilities of the neural network. The layer thickness, set to 0.1 mm, and the hatch distance, set to 0.2 mm, are kept constant. Alternating meander scanning is applied as the underlying exposure strategy. In contrast to the generation of training data, the powder bed temperature is set to 170 °C. The powder bed temperature is specified according to a preceding optimization of the powder bed temperature to avoid curling due to varying exposure parameters, displayed in Table 6. Varying scan speeds and energy densities are applied to enable the validation of the generalization capability for various processing parameters that do not occur in the training dataset.

**Figure 6:**
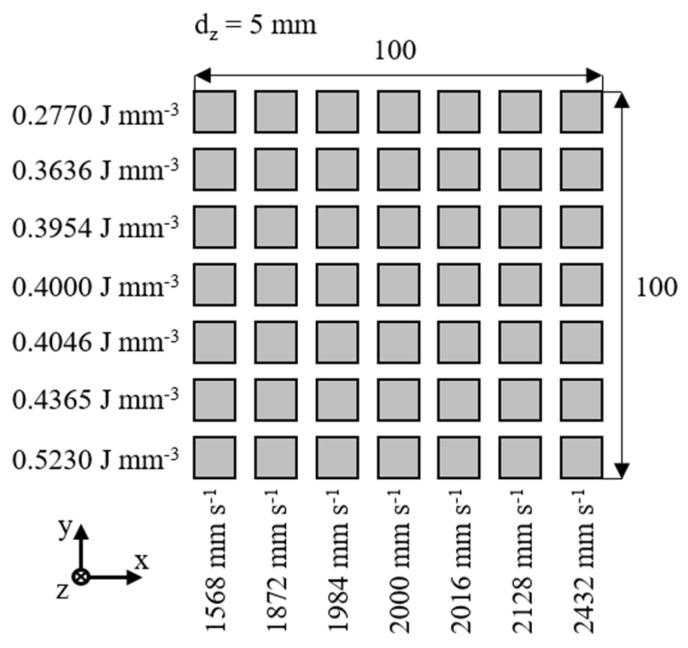
Spatial process layout for the validation of the trained neural network.

Based on the energy density variation, the corresponding laser power is determined for each factor combination based on the scan speed, the underlying specified energy density and constant boundary conditions. The validation layout compromises a 7^2^ full factorial design of experiment, displayed in Figure 6. Therefore, 49 distinct parameter combinations are applied to validate the generalization capabilities of the trained neural network.

**Figure 7:**
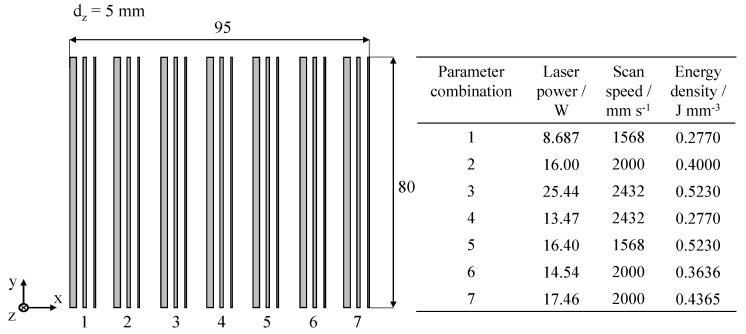
Graphical depiction of the build process layout for the validation of thin-walled geometries, illustrating the applied fractional factorial design of experiments.

In addition to the exposure of square cross-sections, the influence of thin-walled geometries on the prediction accuracy of the neural network is assessed by manufacturing components with a wall thickness of 2 mm, 1 mm, and 0.5 mm, respectively. Virgin Polyamide 12 is used as the underlying material. The applied process layout and corresponding parameters for the validation of thin-walled samples are displayed in Figure 7.

Validating the prediction accuracy of thin-walled samples is based on a fractional factorial design of experiments that includes the variation of the wall thickness and the applied processing parameters. Therefore, the applied processing parameters allow for considerably increasing the range of occurring process states, enabling the validation of the trained neural network.

### 2.6. Explanation of the Underlying Regression

The explanation of underlying mechanisms of neural network-based modeling is based on the concept of “Shapley Additive Explanation”. The applied concept, introduced by Lundberg and Lee (2017) [55], allows for determining the contribution of discrete optical input values to the resulting porosity value. Due to the architecture of the applied neural network and the underlying training dataset, “Deep SHAP” is applied to determine the contribution of distinct values of exemplary optical time series on the predicted part-specific porosity.

## 3. Results and Discussion

### 3.1. Process-Integrated Measurement of Optical Melt Pool Properties

**Figure 8:**
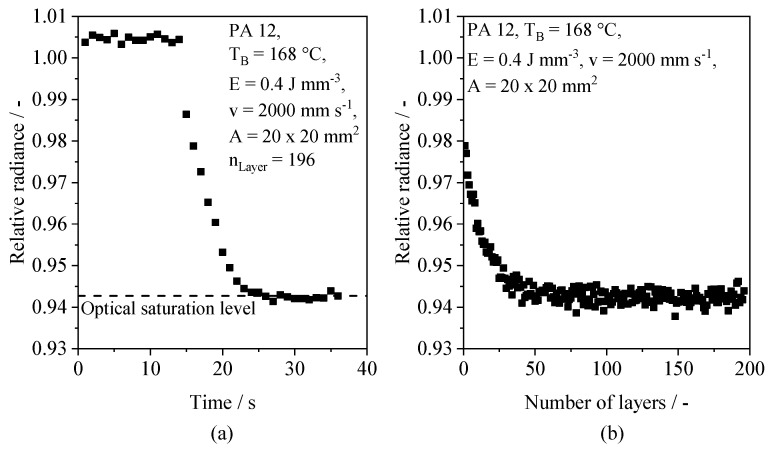
(**a**) Time-resolved evolution of detected part-specific relative radiance; (**b**) Influence of the number of consecutive layers on the emerging saturation level.

The time-resolved measurement of varying optical melt pool properties, specifically of the detected radiance relative to unexposed powder bed sections, constitutes the foundation for the subsequent deep learning-based regression. With regard to process- and material-specific influences, as well as time dependencies, an implicit representation of varying process conditions can be observed. The relative radiance, corresponding to the normalized radiance of visible light detected for a particular area, is applied as the underlying metric for quantifying the time-dependency of optical properties of the melt pool. PBF of Polyamide 12 can be characterized by a time dependency of optical melt pool properties within discrete layers and a dependency of optical melt pool properties of the number of consecutive layers, displayed in Figure 8. Within each layer, a monotonous temporal decrease in the part-specific relative radiance can be observed. The time-resolved evolution is characterized by the convergence towards a process-specific level of the relative radiance, referred to as the optical saturation level. Considering the layer-wise build process, the layer-specific optical saturation level displays a dependency of the number of consecutive layers, thus indicating an influence of previously exposed layers on optically represented process properties.

**Figure 9:**
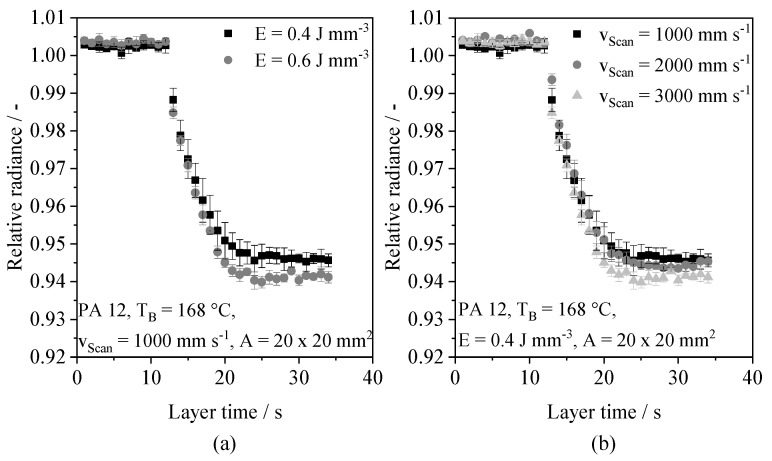
Influence of the applied energy density (**a**) and the applied scan speed (**b**) on the time-resolved optical decay, z = 19.9 mm, n = 3.

Considering the applied process parameters, a constant saturation level is formed after exceeding a number of 40 consecutive layers, and a building height of 4 mm, respectively. The influence of the number of layers on the optical saturation level can be correlated with findings by Greiner et al. (2021) [56], showing a layer-dependent increase in the temperature gradient of the unexposed part cross-section relative to the surrounding powder bed surface. Therefore, optical melt pool properties can be correlated with thermal process properties. In addition to the influence of the number of consecutive layers, varying process parameters are correlated with varying time-resolved optical melt pool properties, indicating an influence on the optical saturation level, displayed in Figure 9.

**Figure 10:**
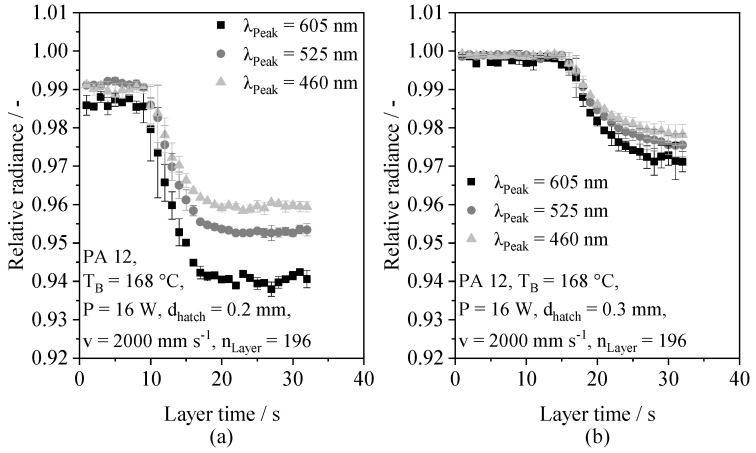
Illustration of wavelength-specific time-resolved optical melt pool properties for an applied hatch distance of 0.2 mm (**a**) and 0.3 mm (**b**), respectively, n = 3.

With regard to the observed variance of optical melt pool properties, merely insignificant correlations of the optical saturation level and the applied process parameters can be observed. Accordingly, the observed inherent variance restricts the possibility of applying regression models based on static values, such as the optical saturation level, for the in situ prediction of part properties based on time-resolved optical melt pool properties. The observed influence of both the applied energy density and the exposure speed can be correlated with increased melt temperatures [8], indicating an influence of thermal process conditions on optical melt pool properties. Based on the distinction of wavelength ranges, structural parameter-dependent deviations in the wavelength-dependent temporal decay are observed, shown in Figure 10.

An increased energy input, displayed in Figure 10a, is correlated with a reduced measured radiance of long-wave radiation compared to short-wave radiation, thus implying a positive correlation of the wavelength and the monitored relative decay. In contrast, merely insignificant influences of the monitored spectrum can be observed for the application of an insufficient energy density level, shown in Figure 10b. With regard to the wavelength-dependent analytic modeling of light scattering in semi-crystalline polymers by Molnár et al. (2020) [57], the observed wavelength dependencies can be correlated with the occurrence of Mie scattering inside the melt pool. Therefore, wavelength-dependent deviations are associated with varying concentrations and geometries of spherulites, defects and pores inside the melt pool. The occurrence of scattering, influenced by the presence of optically inhomogeneous sections, provides a possible explanation for the previously observed influence of preceding layers on observed optical properties. With regard to the temporal decay, the occurrence of scattering centers exhibits a time dependency, thus compromising additional information on the coalescence process, the melting of spherulites and the time-dependent occurrence of defects. Consequently, the obtained results indicate an influence of process states, such as excessive or insufficient energy input, on resulting wavelength-dependent optical melt pool properties. Therefore, the influence of processing parameters and material properties on the part-specific time series of optical melt pool characteristics represents the physical foundation of the regression.

### 3.2. Influence of the Network Architecture on the Prediction Accuracy

**Figure 11:**
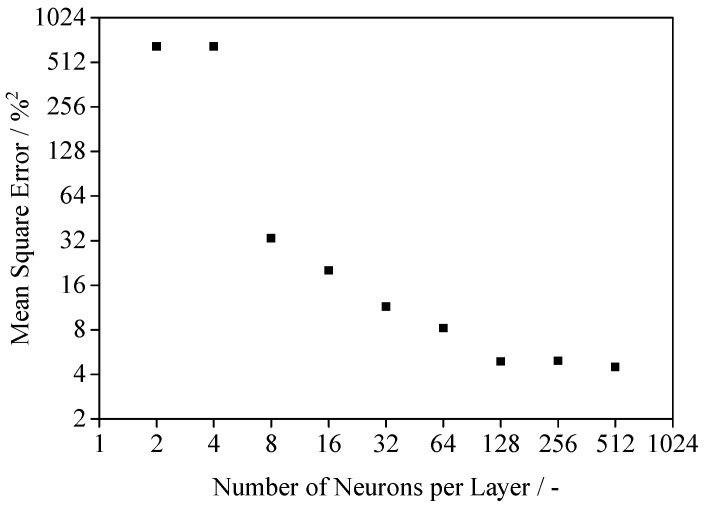
Dependency of the mean prediction error and the width of the network, standard deviation not visible.

The optimization of the underlying architecture of the neural network is of essential importance for the subsequent modeling of the regression. Therefore, varying network architectures are assessed based on the generalization error of the network. The relative mean square error, corresponding to the number of neurons within the block structure, is displayed in Figure 11.

The observed decay indicates a positive correlation of the obtained precision and the applied number of neurons. Satisfactory modeling can be observed with the number of neurons surpassing n = 128. However, a convergence of the modeling error can be observed with an increasing width of the block structure of the neural network. With regard to the influence of the network architecture on the generalization error, Yang et al. (2020) [58] describe the influence of the width of the network in the context of the bias-variance-dilemma. By increasing the number of neurons per layer, both variance and bias can be reduced, leading to an improved generalization of the neural network. Consequently, a width of 512 neurons per layer is suitable for modeling the present regression while limiting required computational resources. Consequently, subsequent modeling is based on a neural network exhibiting a layer width of n = 512 neurons.

### 3.3. Validation of the Trained Neural Network

**Figure 12:**
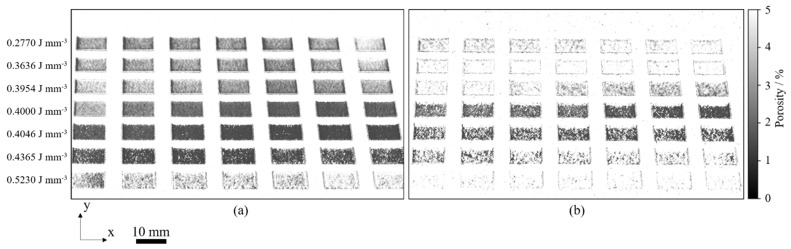
Predicted, spatially resolved cumulative porosity distribution obtained for the validation build process for the application of virgin (**a**) and pre-aged (**b**) PA 12 powder.

The quality and suitability of the presented neural network with regard to process monitoring applications substantially depend on the capability to model process conditions that are not included in the training data. Preceding the validation step, the neural network is trained using the experimentally obtained training data. In contrast to the training of the neural network, the validation of the trained model compromises the pre-defined validation process layout and the application of varying material properties. Figure 12 displays the spatially resolved output provided by the neural network for the application of virgin (a) and pre-aged (b) Polyamide 12 powder, representing the mean value of n = 50 layers.

**Figure 13:**
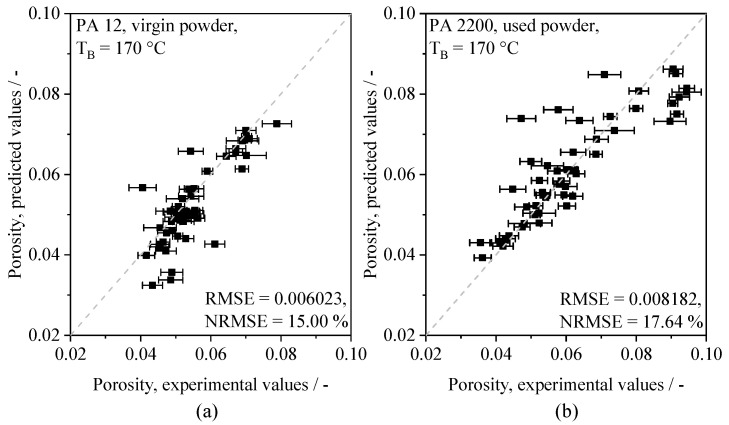
Relation of experimental and predicted, part-specific porosity values for the use of virgin (**a**) and pre-aged (**b**) PA 12 powder, perfect fit indicated by dashed line.

The resulting two-dimensional matrix of spatially resolved porosity values displays the influence of varying processing parameters on the formation of defects in the core region and in the edge region that significantly contribute to the porosity of a particular cross-section. In accordance with findings by Pavan et al. (2017) [6], the modeled spatial porosity distribution indicates a predominant influence of the variation of the applied energy density and the aging state of the used material, evident in Figure 12. Based on the validation process layout, a satisfactory accordance of predicted, part-specific porosity values and corresponding experimental values can be observed, displayed in Figure 13.

The relation of predicted and experimentally obtained values indicates the general capability of the proposed approach to model the regression of time-resolved optical melt pool properties and resulting part porosity. In contrast to the use of pre-aged powder, a slight, part-specific underestimation of predicted cumulative porosity values can be observed for the application of virgin powder, specifically for near-optimum parameter combinations. With regard to the application of varying cross-sections represented in the training dataset and the validation process layout, respectively, an influence of geometry-dependent temperature fields [8], significantly differing from larger cross-sections, needs to be considered. In addition to geometric influences, statistical deviations of predicted and experimentally assessed values, correlated with the application of aged Polyamide 12, indicate an influence of physical material properties on the prediction accuracy. When applying the neural network on the manufacturing of aged powder, an increase in the overall predicted variance and the average prediction error can be observed. However, the increased variance of the part-specific porosity, induced by the use of aged powder, can be modeled successfully. In addition to the influence of the material aging state, an influence of part-specific layer times can be observed. With regard to the applied process layout, the applied energy density exhibits a negative correlation with part-specific layer times. Therefore, resulting porosity values can be interpreted as a superposition of processing parameters and the part-specific layer time.

**Figure 14:**
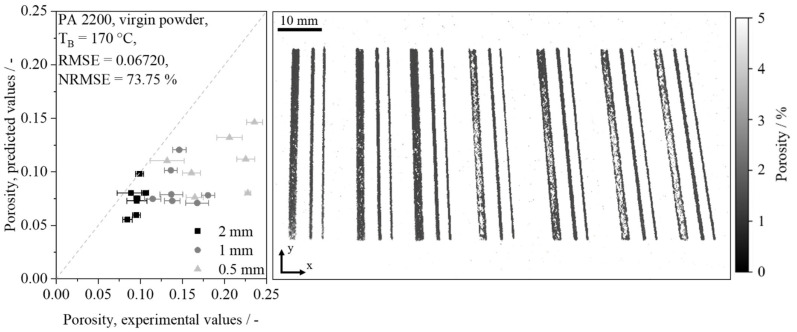
Illustration of the relation of predicted and experimentally assessed values for the production of thin-walled components depending on wall thickness using virgin Polyamide 12, perfect fit indicated by dashed line.

Considering the predominant accordance of experimental and predicted values, a generalization concerning varying physical material properties can be achieved. A further aspect of the generalization capability of the network concerns the influence of the chosen spatial resolution. Given the low range of underlying geometric shapes represented in the training data, the accordance with locally resolved values indicates the capability of the neural network to predict porosity values over a wide range of spatial resolutions. With regard to results presented by Zhang et al. (2017) [39], who demonstrated the ability of in-process image-based porosity quantification for the processing of metal alloys with a root mean square error (RMSE) of 1.32%, root mean square errors of 0.6023%, and 0.8182%, obtained for virgin and aged powder, are considered satisfactory. The validation of thin-walled samples concerns the demonstration of the capability of the network to generalize on geometries varying in a broad range. Considering the observed deviations between predicted and experimentally assessed values, a considerably increased prediction error is observed for the porosity prediction of thin-walled samples, displayed in Figure 14.

The authors assume that the observed deviations may occur due to a sparse training dataset, exclusively compromising cuboid samples, and deviations concerning the experimental measurement of thin-walled validation samples due to an increased surface-volume ratio. In addition to a limited representation of thin-walled samples, further influences on the obtained prediction accuracy may include the occurrence of optical measurement errors due to a potentially limited optical traceability of irregularly distributed, superposed voids and a potential scale dependency of the coalescence of thin-walled specimens. The experimentally assessed elevated level of porosity, which is not represented in the training dataset, can be correlated with findings of Wörz et al. (2019) [2], describing a negative correlation between the wall thickness and experimentally assessed porosity levels. With regard to formed morphologies, Sindinger et al. (2020) [5] describe a shell-core structure of thin-walled specimens, derived using computed tomography. Similar parameter-depending shell-core structures can be observed for a sample width of 2 mm, partially displaying dense part contours and elevated porosity levels inside the part. Considering the structural accordance of neural network-based prediction with findings described by Sindinger et al. (2020) [5], the prediction error, increasing with a decreasing sample width, is assumed to partially rely on surface effects, thus affecting the experimental measurement of thin-walled samples due to the influence of surface roughness. In addition to the influence on the experimental porosity measurement, an influence of surface effects on the part-specific assessment of the predicted porosities is considered relevant due to a limited spatial resolution of the applied camera setup of 0.04 mm. Therefore, the occurrence of surface effects indicates the necessity of increasing the spatial resolution of the monitoring setup and applying computed tomography for assessing the prediction accuracy of thin-walled samples in future work. In addition to previously discussed influences, the relation of the applied resolution and the average particle size indicates an influence of the powder bed topology on predicted porosity values of thin-walled samples.

In spite of the high variance of experimentally obtained porosity values in the validation step, the satisfactory modeling of structural defects can be demonstrated. Furthermore, morphologies that are described specifically for thin-walled samples [5] can be modeled successfully for unknown parameter combinations and unknown geometries. Consequently, a generalization of the modeling approach on a part-specific level and on a mesoscopic scale, thus enabling the inline monitoring of local defects, can be observed. Despite the demonstrated modeling of local defects, further advances are required in future research to increase the prediction accuracy of the applied approach to allow for the precise prediction of thin-walled components.

### 3.4. Numerical Contribution of Input Values on the Regression

The explanation of the underlying mechanisms of neural-network-based modeling is significant to assess the reliability of the underlying regression [59]. Given the complex nature of the underlying processes and considering the structural complexity of the artificial neural network, the concept of Shapely Additive Explanation, proposed by Lundberg and Lee (2017) [55], is applied. By applying SHAP, specifically in the form of deep SHAP, the contribution of distinct optical input values on the scalar porosity value can be derived, thus enabling insights into the modeling of the regression.

**Figure 15:**
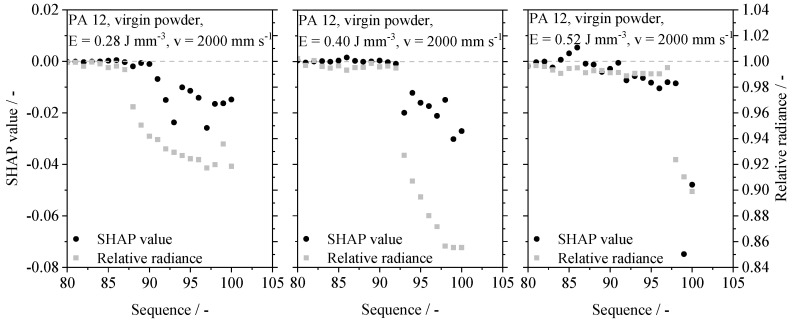
Numerical contribution of distinct part-specific time steps within the time series on the resulting predicted part porosity, time steps 80 to 100 displayed.

The application of SHAP on exemplary datasets indicates the mutual dependence of the optical input values and the resulting contribution on the obtained porosity value. Displayed in Figure 15, the underlying regression implicitly adapts the influence of input values relevant for the regression. When considering the application of near-optimal process parameters, input values preceding the exposure process do not significantly affect predicted part properties. However, an increase, as well as a decrease in the applied energy density lead to the formation of structural differences concerning the contribution of distinct time steps. A reduction in the applied energy density is correlated with reduced numerical contributions of the initial decay of the melt pool radiance. In contrast, the neural network exhibits a significant contribution of discrete time steps preceding the exposure process when exceeding the optimum energy density. This contribution can be correlated with an optically represented, superficial melting of unexposed powder particles due to the increased energy input into the melt pool.

Considering the applied process layout, increased energy density values are correlated with reduced part-specific layer times. Therefore, the divergent mechanisms determining the part-specific porosity values are expected to not exclusively rely on divergent processing parameters. However, depending on the applied process parameters, structural deviations in the contribution of distinct time steps on the resulting part property, derived by the neural network, are observed. The parameter-dependent contribution of distinct time steps on the resulting porosity value indicates the implicit distinction of divergent process states. The recognition of specific process states is considered essential for enabling the previously demonstrated ability of the network to model varying part-specific porosity values, influenced by variations of process parameters and material aging states. Consequently, underlying mechanisms for the in situ prediction of part-specific porosity values compromise the implicit recognition of superposed process influences.

## 4. Conclusions and Outlook

A novel approach for quantifying part porosity in the powder bed fusion of polymers based on optical data, assessed in situ, could be demonstrated within the present paper. The presented approach applies a deep residual neural network that allows for the process-integrated porosity analysis of PBF-LB/P based on the analysis of time series. Temporal changes in optical properties of exposed powder bed sections, representing the input data for the monitoring approach, are characterized by a quasi-monotonous, temporal decay of the radiance of the melt pool, correlated with a time dependency of the occurrence of scattering centers within the melt pool. Varying processing parameters lead to a variation of the time-resolved anisotropic radiance of the melt pool, representing the physical foundation of the subsequent regression, hence allowing for the determination of component properties both on component-specific and mesoscopic scales. The successful inline modeling of the part porosity could be demonstrated for the application of divergent processing parameters, part geometries, and the use of aged Polyamide 12 powder. By determining the influence of distinct optical input values on the corresponding porosity value, the predominant influence of the whole consolidation phase on the derived value was identified. Furthermore, the observed parameter-dependency of the numerical contribution of distinct time steps indicates the implicit distinction of process states, such as insufficient and excessive energy input into the melt. Future work will include enhancing the prediction accuracy and the robustness towards divergent geometries, materials, and processing parameters by enlarging the training dataset. Furthermore, the application of the demonstrated regression network in the real-time process monitoring and the automated, material-specific identification of optimized process parameters embeds relevance for future industrial applications. Considering the possibility of assessing the time-resolved evolution of part properties by determining the resulting part properties at distinct time steps, the proposed approach embeds a novel concept for enhancing the understanding of the consolidation step.

Consequently, the presented deep learning-based approach represents the foundation for various applications, including the enhanced understanding of parameter interactions and the vastly accelerated development of novel materials in polymer-based powder bed fusion by means of in-process optimization of process parameters.

## Figures and Tables

**Table 1:** Physical properties of virgin Polyamide 12 powder.

Physical Property	Value
Viscosity number	55 mL g^−1^
Melting peak	179 °C
Crystallization peak	152 °C

**Table 2:** Physical properties of pre-aged Polyamide 12 powder.

Physical Property	Value
Viscosity number	80 mL g^−1^
Melting peak	178 °C
Crystallization peak	150 °C

**Table 3:** Parameters and properties of the applied camera assembly.

Property	Value
Average spatial resolution	0.04 mm
Focal length	50 mm
Pixel pitch	1.55 µm
Sensor area	6.287 × 4.712 mm^2^
Aspect ratio	4:3
f-number	f/8
Exposure time	66.6 ms
Exposure index	ISO 100
IR filter	Hoya CM500
Frame rate	1 Hz

**Table 4:** Processing parameters applied for the generation of training data.

Part Label	Laser Power/W	Scan Speed/mm s^−1^	Hatch Distance/mm	Energy Density/J mm^−3^
1	8	1000	0.2	0.400
2	16	2000	0.2	0.400
3	24	3000	0.2	0.400
4	12	1000	0.2	0.600
5	24	2000	0.2	0.600
6	8	1000	0.3	0.266
7	16	2000	0.3	0.266
8	24	3000	0.3	0.266
9	8	1000	0.15	0.533
10	16	2000	0.15	0.533
11	24	3000	0.15	0.533
12	24	4000	0.15	0.400
13	12	4000	0.075	0.400
14	8	4000	0.075	0.266
15	8	1000	0.2	0.400
16	16	2000	0.2	0.400
17	24	2000	0.2	0.600
18	24	4000	0.15	0.400
19	24	2000	0.2	0.600
20	16	2000	0.2	0.400

**Table 5:** Overview of the boundary conditions and hyperparameters applied for training the artificial neural network.

Property	Value
Optimizer	Adaptive Moment Estimation
Initial learning rate	0.0002
Exponential learning decay rate	10^−6^
First momentum decay rate β_1_	0.95
Second momentum decay rate β_2_	0.999
Number of training iterations	50,000
Loss function	Mean Square Error
Dropout rate	0.125
Layer weight initialization	Normal randomized distribution, σ = 0.05

**Table 6:** Overview of exposure parameters applied for the network validation.

Scan Speed/mm s^−1^	Energy Density/J mm^−3^
1568	0.2770
1872	0.3636
1984	0.3954
2000	0.4000
2016	0.4046
2128	0.4365
2432	0.5230

## Data Availability

The data presented in this study are available on request from the corresponding author. The data are not publicly available due to ongoing research in this field.
